# Supplementary material for: Development of a Live‐Cell Imaging Assay to Elucidate Spatiotemporal Dynamics of Extracellular Vesicle Fusion with Target Cells
Source: J Extracell Vesicles. 2026 Mar 1;15(3):e70228. doi: 10.1002/jev2.70228 (PMC12949999; doi:10.1002/jev2.70228)
Supplement: Supplementary file 1 — Supporting Figures 1–13: jev270228‐sup‐0001‐figures.pdf [file JEV2-15-e70228-s006.pdf]

S1

A

fraction number	1	2	3	4	5	6	7	8	9	10	11
density (g/mL)	1.324	1.207	1.180	1.157	1.130	1.103	1.083	1.063	1.043	1.029	1.012

B

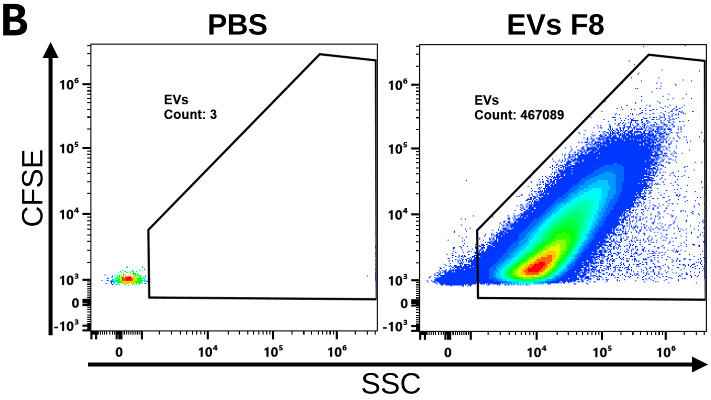

C

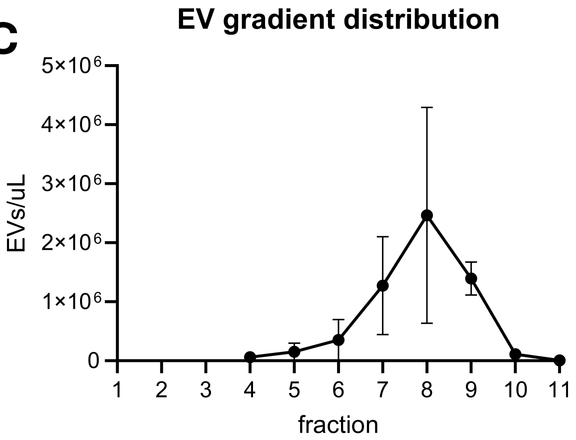

S2

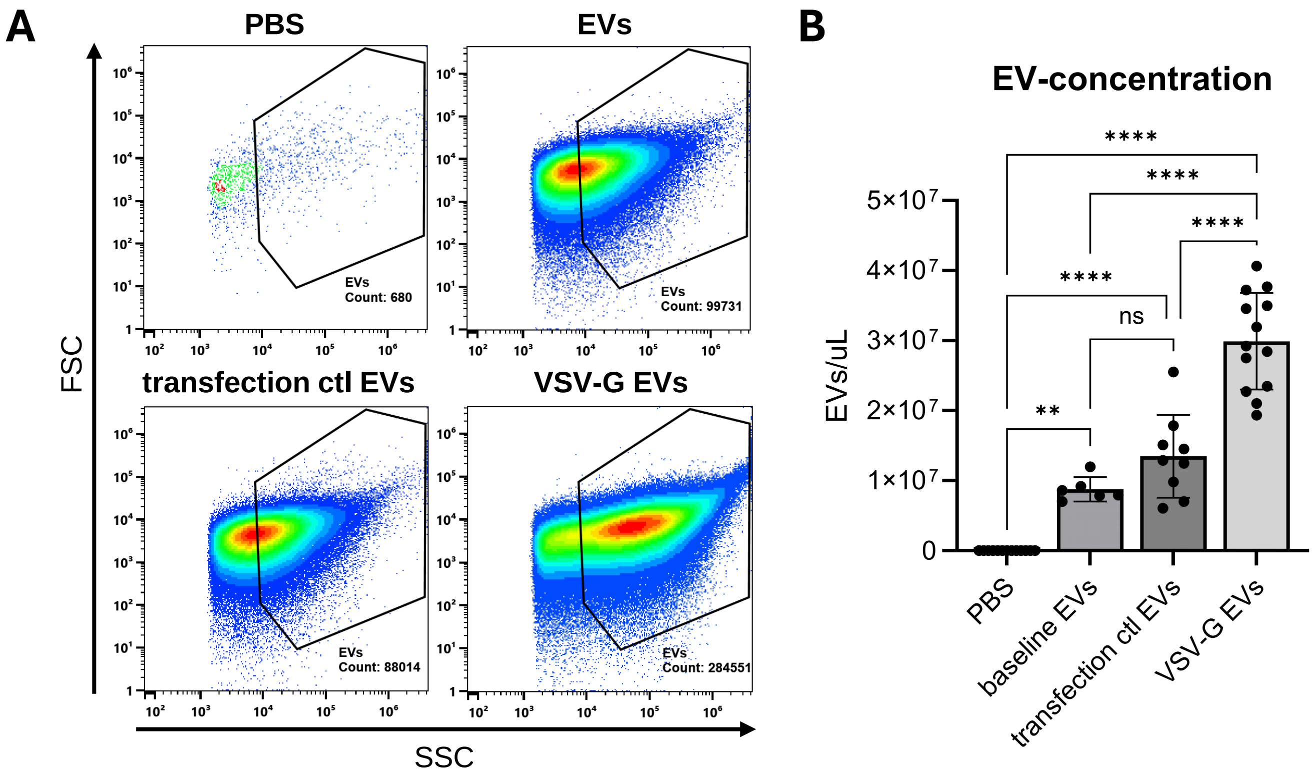
A
PBS
EVs
transfection ctl EVs
VSV-G EVs
EVs
Count: 680
EVs
Count: 99731
EVs
Count: 88014
EVs
Count: 284551
FSC
SSC
B
EV-concentration
EVs/uL
5×10⁷
4×10⁷
3×10⁷
2×10⁷
1×10⁷
0
****
****
****
****
ns
**
PBS
baseline EVs
transfection ctl EVs
VSV-G EVs

S3

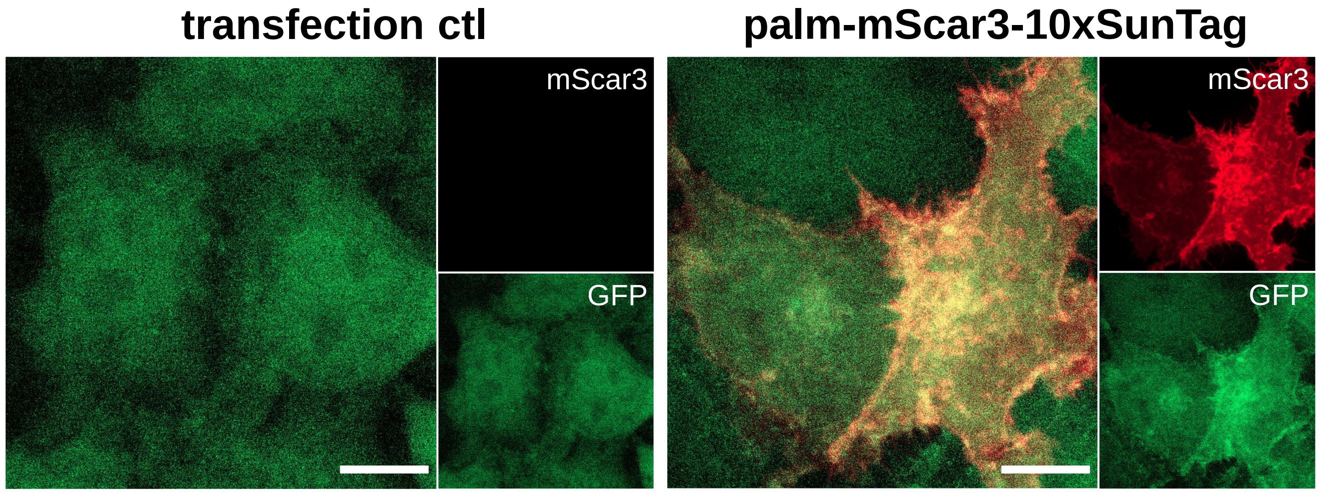
transfection ctl
palm-mScar3-10xSunTag
mScar3
GFP
mScar3
GFP

S4

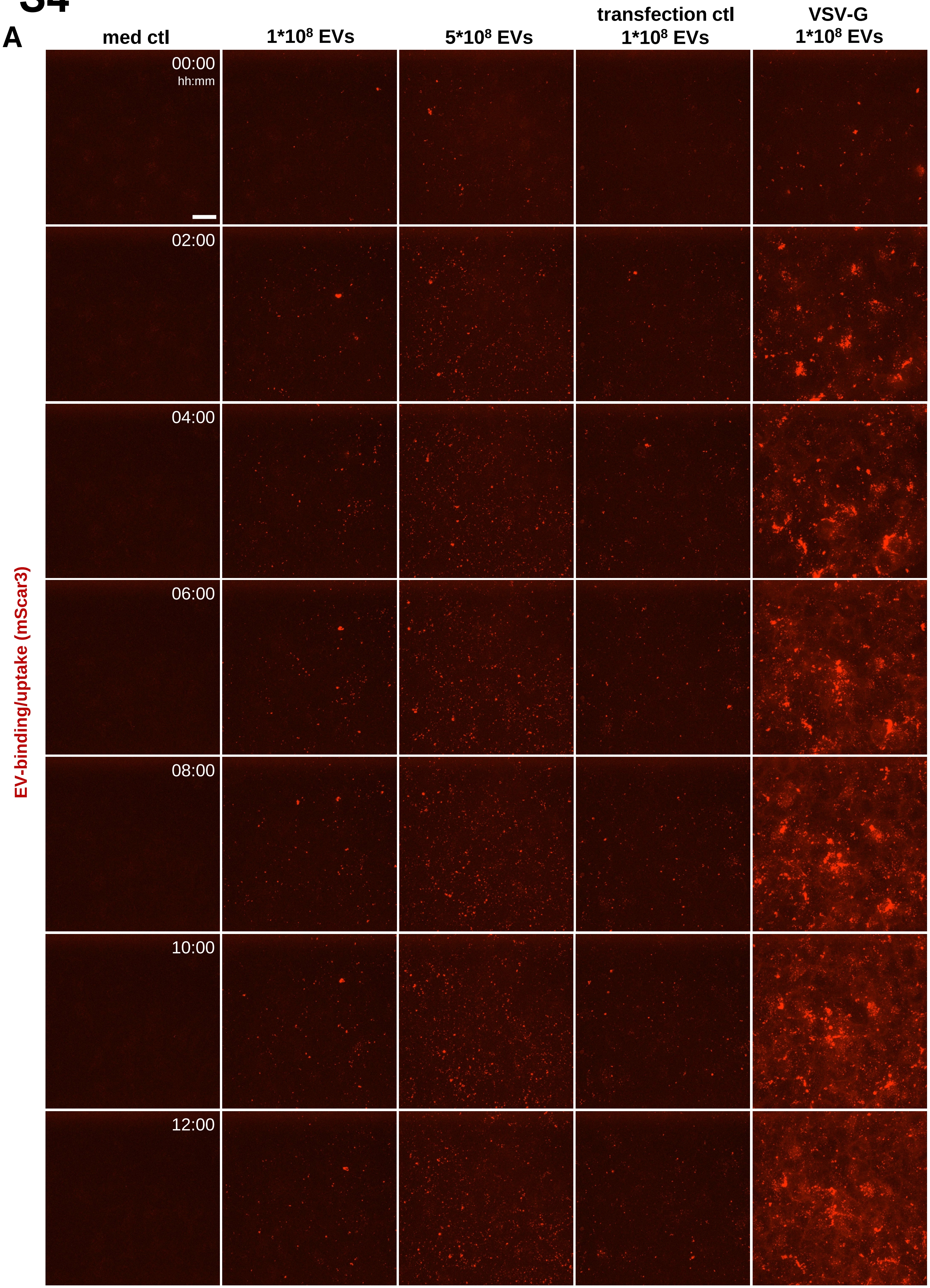
A
med ctl
1*10^8 EVs
5*10^8 EVs
transfection ctl
1*10^8 EVs
VSV-G
1*10^8 EVs
00:00
hh:mm
02:00
04:00
06:00
08:00
10:00
12:00
EV-binding/uptake (mScar3)

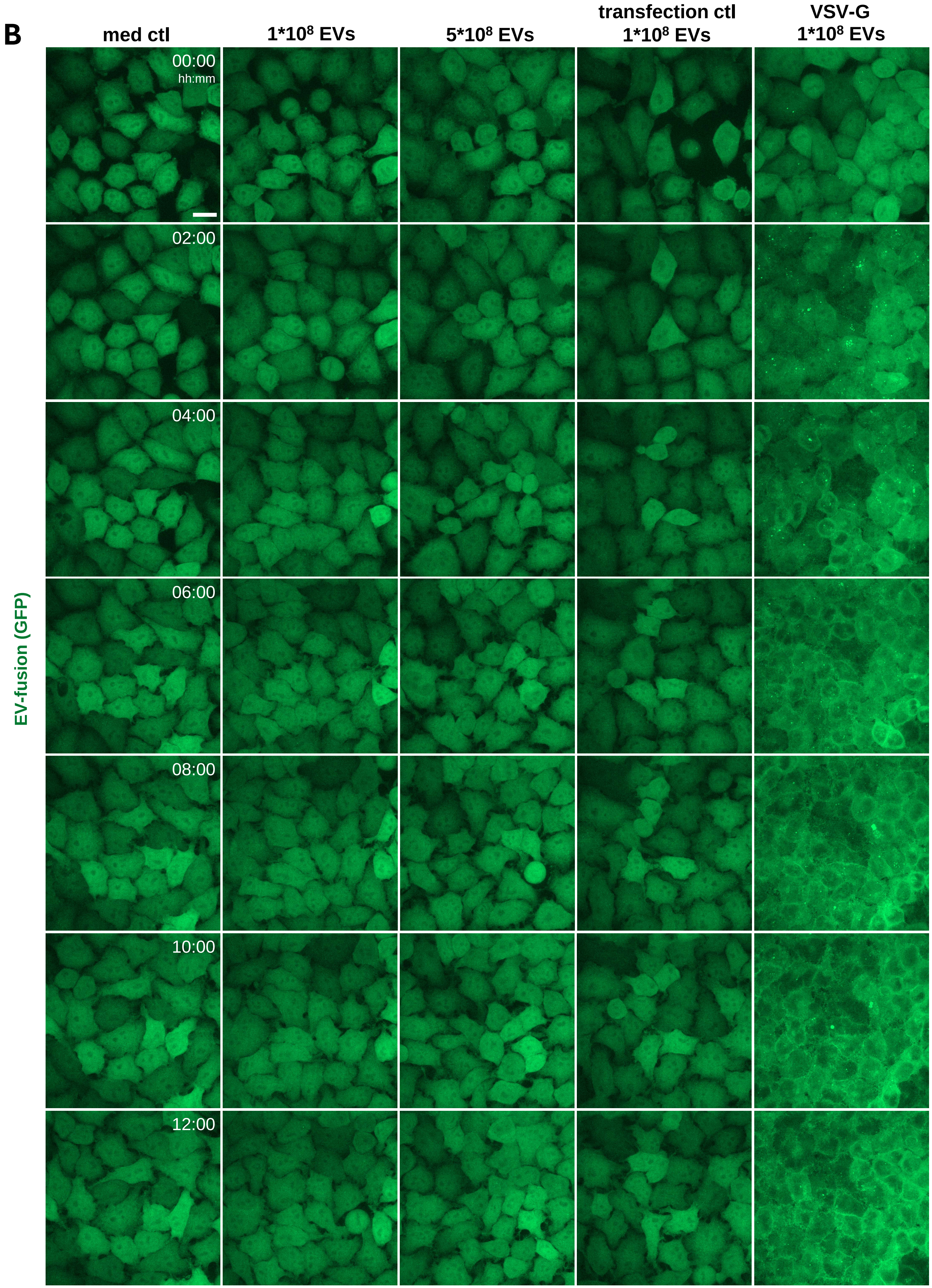
B
med ctl
1*10^8 EVs
5*10^8 EVs
transfection ctl
1*10^8 EVs
VSV-G
1*10^8 EVs
00:00
hh:mm
02:00
04:00
06:00
08:00
10:00
12:00
EV-fusion (GFP)

S5

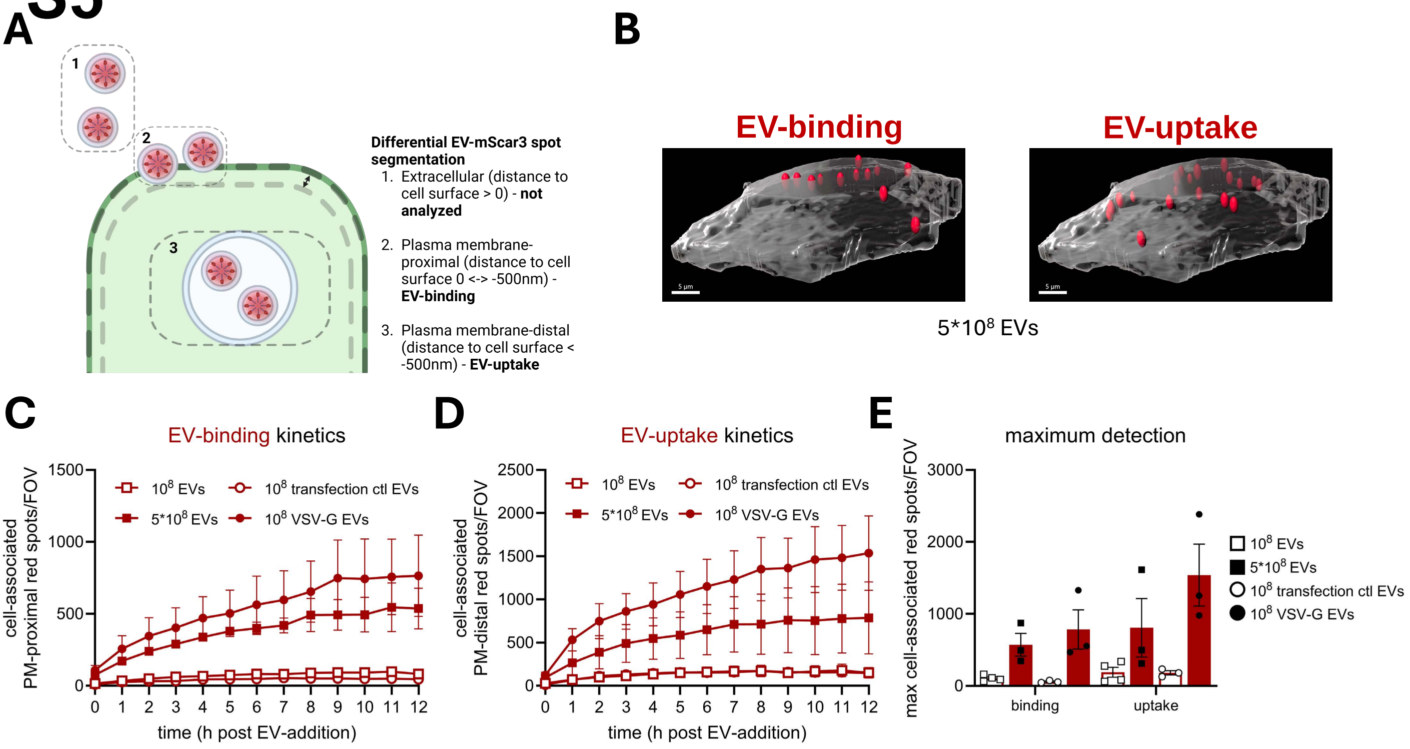
A
1
2
3
Differential EV-mScar3 spot segmentation
1. Extracellular (distance to cell surface > 0) - **not analyzed**
2. Plasma membrane-proximal (distance to cell surface 0 <-> -500nm) - **EV-binding**
3. Plasma membrane-distal (distance to cell surface < -500nm) - **EV-uptake**
B
EV-binding
EV-uptake
5 µm
5 µm
$5*10^8$ EVs
C
EV-binding kinetics
cell-associated PM-proximal red spots/FOV
time (h post EV-addition)
10^8 EVs
$5*10^8$ EVs
10^8 transfection ctl EVs
10^8 VSV-G EVs
D
EV-uptake kinetics
cell-associated PM-distal red spots/FOV
time (h post EV-addition)
10^8 EVs
$5*10^8$ EVs
10^8 transfection ctl EVs
10^8 VSV-G EVs
E
maximum detection
max cell-associated red spots/FOV
binding
uptake
10^8 EVs
$5*10^8$ EVs
10^8 transfection ctl EVs
10^8 VSV-G EVs

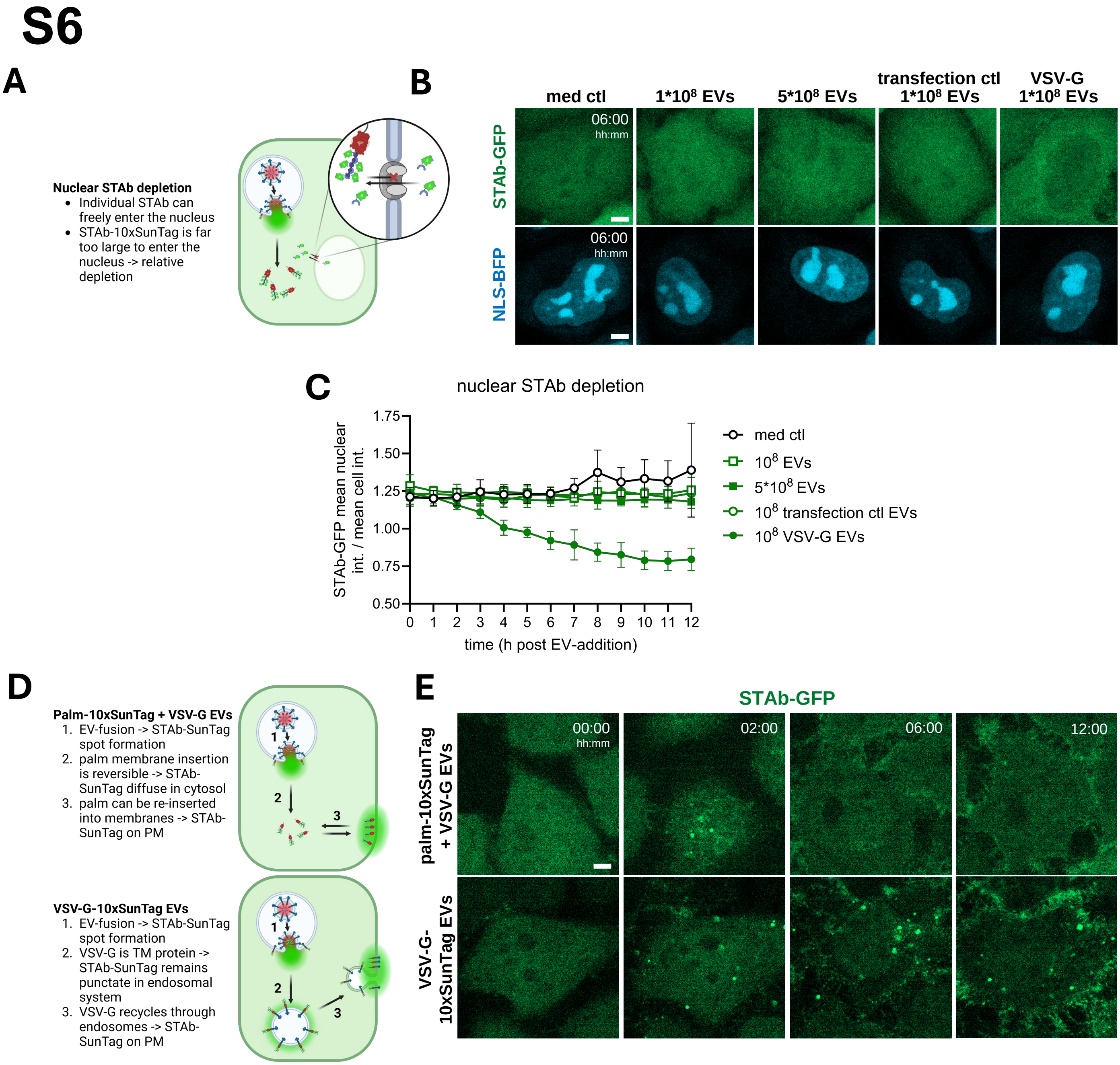
S6
A
Nuclear STAb depletion
• Individual STAb can freely enter the nucleus
• STAb-10xSunTag is far too large to enter the nucleus -> relative depletion
B
med ctl
1*10^{8} EVs
5*10^{8} EVs
transfection ctl 1*10^{8} EVs
VSV-G 1*10^{8} EVs
STAb-GFP
NLS-BFP
06:00 hh:mm
C
nuclear STAb depletion
STAb-GFP mean nuclear int. / mean cell int.
time (h post EV-addition)
med ctl
10^{8} EVs
5*10^{8} EVs
10^{8} transfection ctl EVs
10^{8} VSV-G EVs
D
Palm-10xSunTag + VSV-G EVs
1. EV-fusion -> STAb-SunTag spot formation
2. palm membrane insertion is reversible -> STAb-SunTag diffuse in cytosol
3. palm can be re-inserted into membranes -> STAb-SunTag on PM
VSV-G-10xSunTag EVs
1. EV-fusion -> STAb-SunTag spot formation
2. VSV-G is TM protein -> STAb-SunTag remains punctate in endosomal system
3. VSV-G recycles through endosomes -> STAb-SunTag on PM
E
STAb-GFP
palm-10xSunTag + VSV-G EVs
VSV-G-10xSunTag EVs
00:00 hh:mm
02:00
06:00
12:00

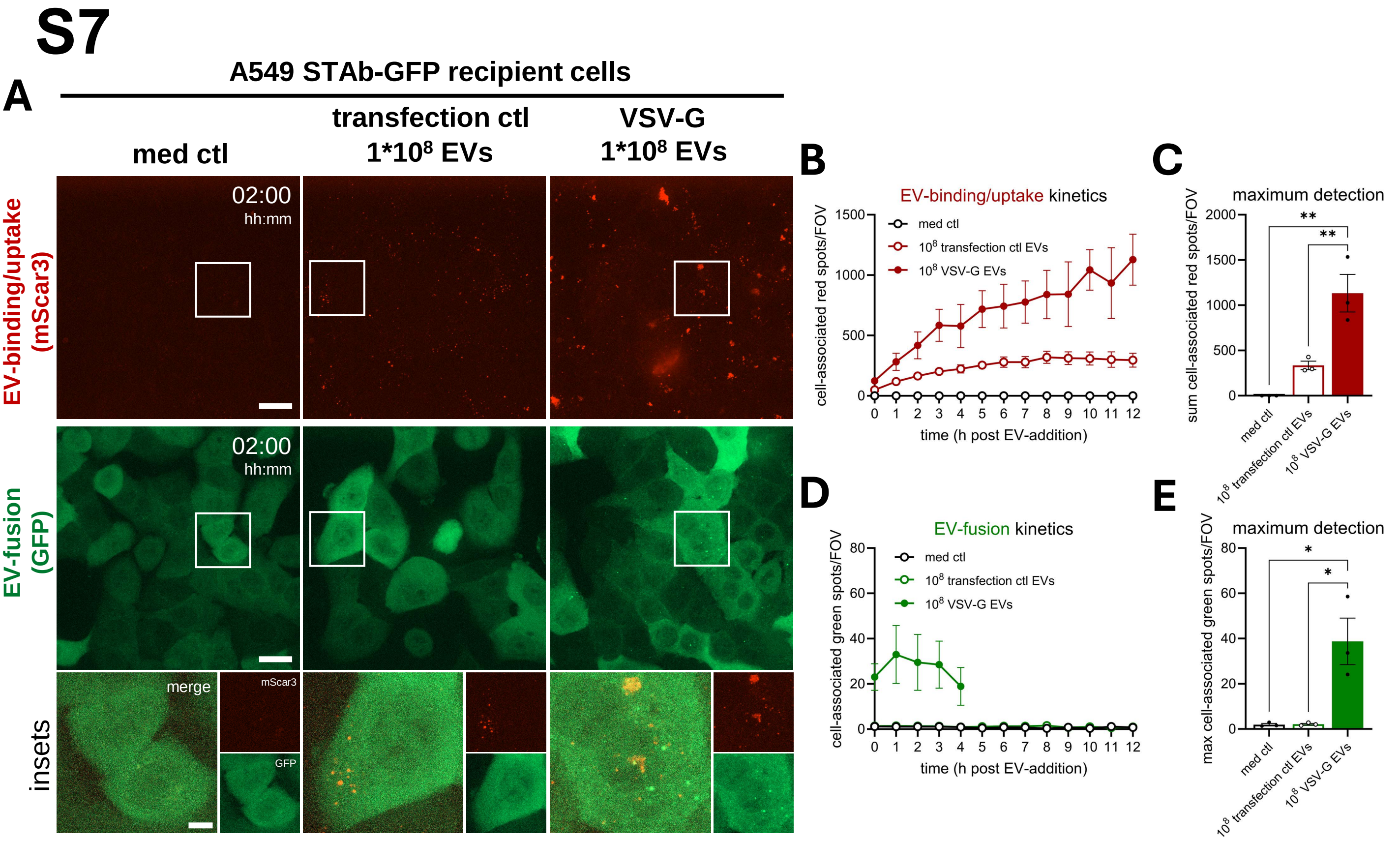
S7
A
A549 STAb-GFP recipient cells
med ctl
transfection ctl
1*10^8 EVs
VSV-G
1*10^8 EVs
EV-binding/uptake
(mScar3)
02:00
hh:mm
EV-fusion
(GFP)
insets
merge
mScar3
GFP
B
EV-binding/uptake kinetics
med ctl
10^8 transfection ctl EVs
10^8 VSV-G EVs
cell-associated red spots/FOV
time (h post EV-addition)
C
maximum detection
sum cell-associated red spots/FOV
med ctl
10^8 transfection ctl EVs
10^8 VSV-G EVs
D
EV-fusion kinetics
cell-associated green spots/FOV
time (h post EV-addition)
E
maximum detection
max cell-associated green spots/FOV

S8

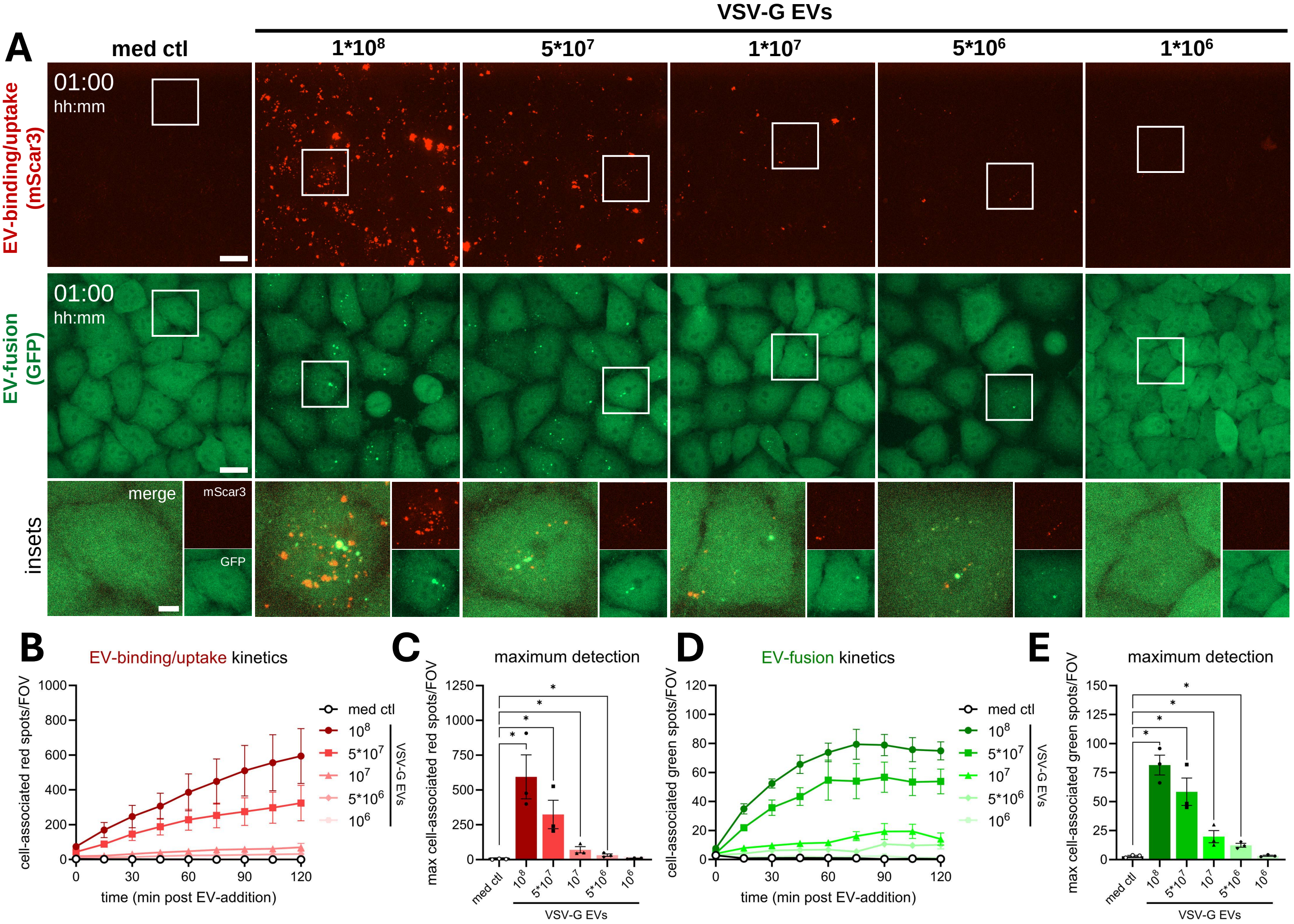

A
VSV-G EVs
med ctl
1*10^8
5*10^7
1*10^7
5*10^6
1*10^6
EV-binding/uptake (mScar3)
EV-fusion (GFP)
01:00 hh:mm
insets
merge
mScar3
GFP
B
EV-binding/uptake kinetics
cell-associated red spots/FOV
time (min post EV-addition)
med ctl
10^8
5*10^7
10^7
5*10^6
10^6
VSV-G EVs
C
maximum detection
max cell-associated red spots/FOV
D
EV-fusion kinetics
cell-associated green spots/FOV
E
max cell-associated green spots/FOV

S9

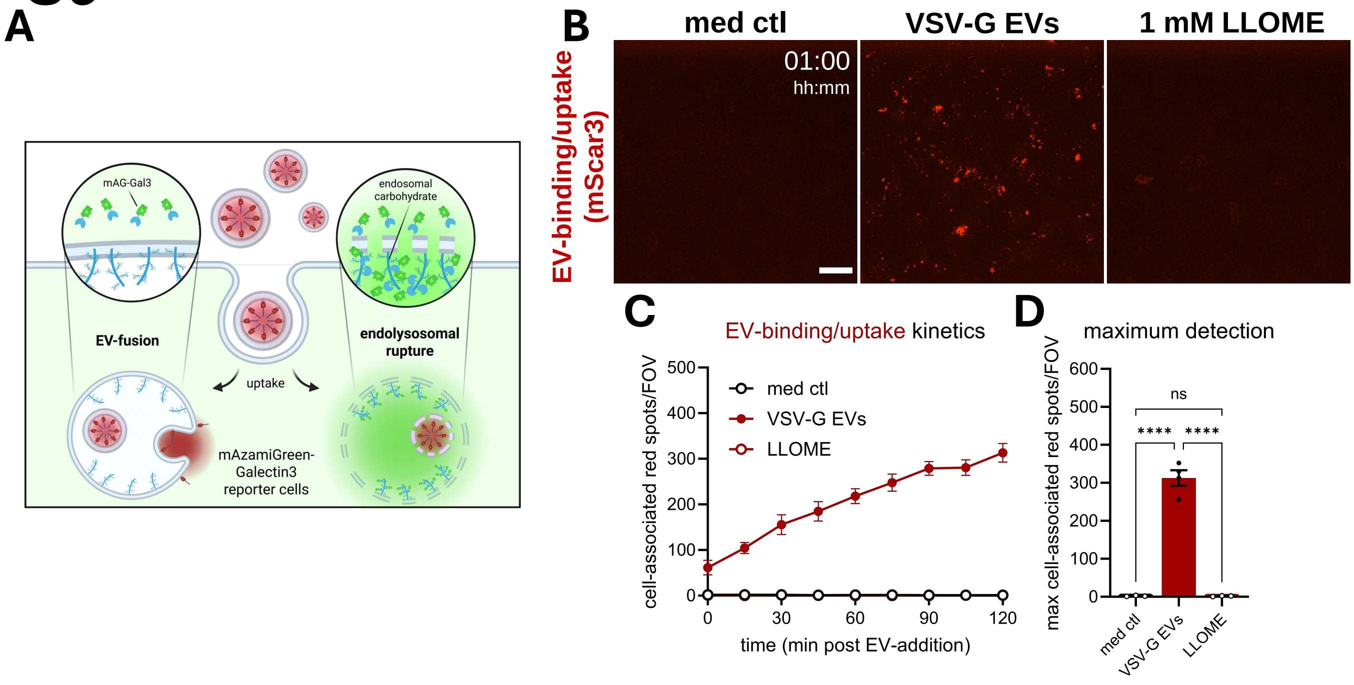
A
mAG-Gal3
endosomal carbohydrate
EV-fusion
endolysosomal rupture
uptake
mAzamiGreen-Galectin3 reporter cells
B
med ctl
VSV-G EVs
1 mM LLOME
01:00
hh:mm
EV-binding/uptake (mScar3)
C
EV-binding/uptake kinetics
cell-associated red spots/FOV
med ctl
VSV-G EVs
LLOME
time (min post EV-addition)
D
maximum detection
max cell-associated red spots/FOV
ns
****
****
med ctl
VSV-G EVs
LLOME

S10

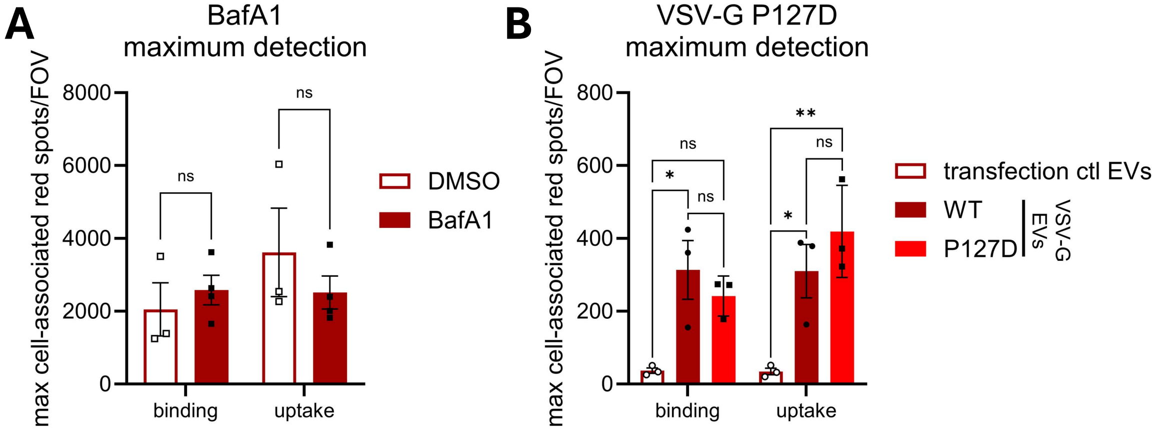
A
BafA1
maximum detection
max cell-associated red spots/FOV
8000
6000
4000
2000
0
ns
ns
binding
uptake
DMSO
BafA1
B
VSV-G P127D
maximum detection
max cell-associated red spots/FOV
800
600
400
200
0
ns
*
ns
**
ns
*
binding
uptake
transfection ctl EVs
WT
P127D
VSV-G EVs

S11

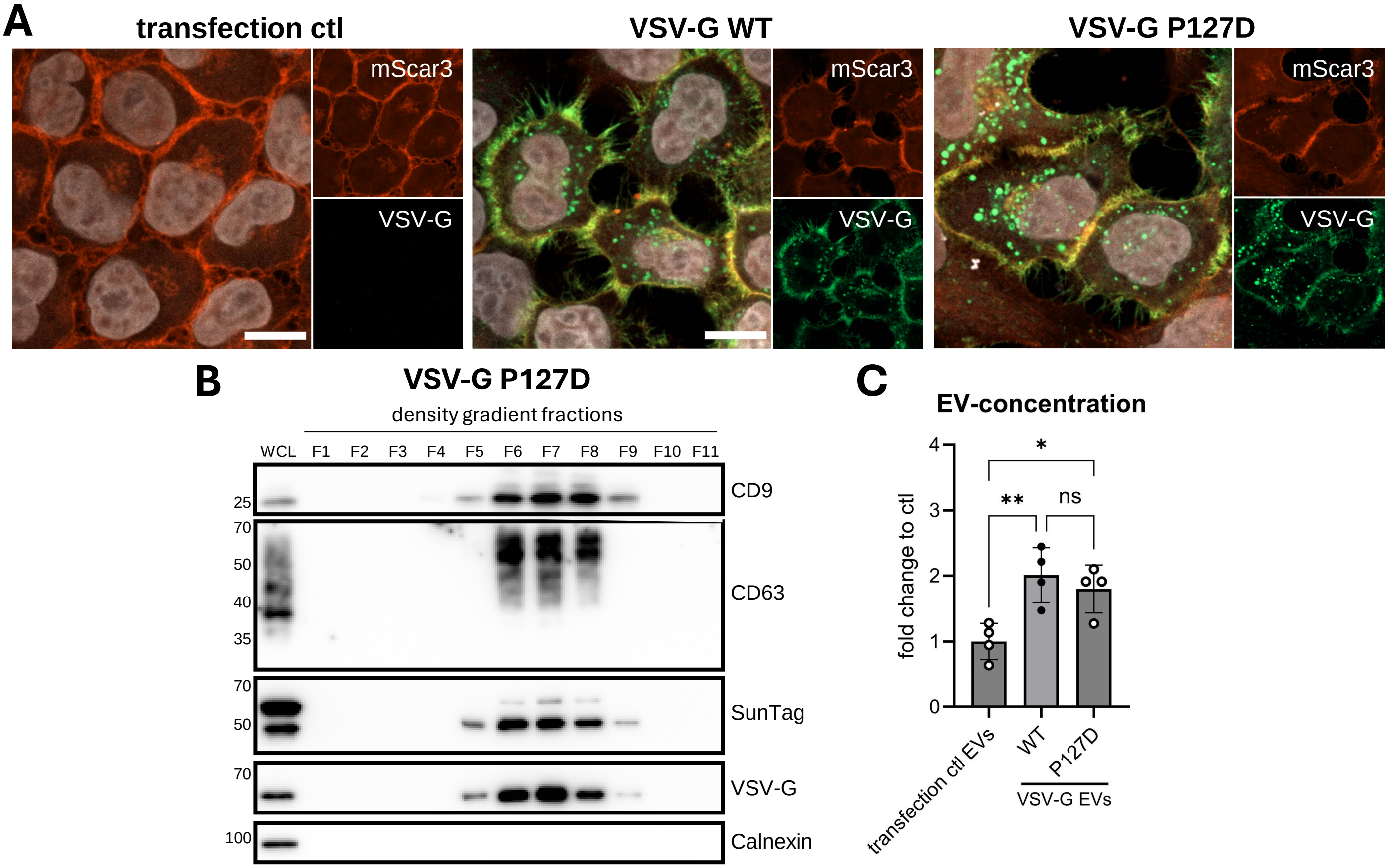
A
transfection ctl
VSV-G WT
VSV-G P127D
mScar3
VSV-G
B
VSV-G P127D
density gradient fractions
WCL
F1
F2
F3
F4
F5
F6
F7
F8
F9
F10
F11
25
70
50
40
35
70
50
70
100
CD9
CD63
SunTag
VSV-G
Calnexin
C
EV-concentration
fold change to ctl
4
3
2
1
0
*
**
ns
transfection ctl EVs
WT
P127D
VSV-G EVs

S12

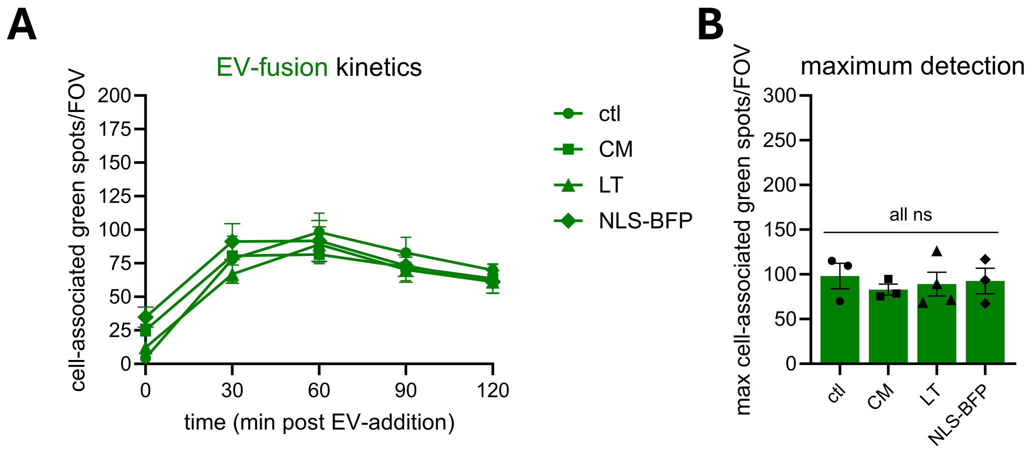
A
EV-fusion kinetics
cell-associated green spots/FOV
200
175
150
125
100
75
50
25
0
0
30
60
90
120
time (min post EV-addition)
ctl
CM
LT
NLS-BFP
B
maximum detection
max cell-associated green spots/FOV
300
250
200
150
100
50
0
all ns
ctl
CM
LT
NLS-BFP
