# Supplementary material for: Development of a Live‐Cell Imaging Assay to Elucidate Spatiotemporal Dynamics of Extracellular Vesicle Fusion with Target Cells
Source: J Extracell Vesicles. 2026 Mar 1;15(3):e70228. doi: 10.1002/jev2.70228 (PMC12949999; doi:10.1002/jev2.70228)
Supplement: Supplementary file 3 — Supporting Table 1: jev270228‐sup‐0003‐Table1.pdf [file JEV2-15-e70228-s003.pdf]

Imaris analysis scripts for object creation

**HeLa STAb cell Surface**	**A549 STAb cell Surface**	**Gal3 cell Surface**	**STEV Spots**	**HeLa STAb Spots**	**Gal3 Spots**	**CellMask Surface**	**LysoTracker Surface**
[Algorithm]<br>Enable Region Of Interest = false<br>Enable Region Growing = true<br>Enable Tracking = true<br>Enable Classify = false<br>Enable Shortest Distance = true<br>[Segmentation Setup]<br>Source Channel Index = 1<br>Enable Smooth = true<br>Surface Grain Size = 0.600 µm<br>[Machine Learning Training]<br>All Channels = false<br>[Split]<br>Region Growing Estimated Diameter = 12.0 µm<br>[Filter Seed Points]<br>"Quality" above 0.0500<br>[Filter Surfaces]<br>"Area" above 330 um^2<br>"Intensity StdDev Ch=1 Img=1" below 4.00<br>"Intensity Mean Ch=1 Img=1" between 1.00 and 8.50<br>[Tracking]<br>Algorithm Name = Brownian Motion<br>MaxDistance = 10.0 µm<br>MaxGapSize = 3<br>Fill Gap Enable = false<br>[Filter Tracks]	[Algorithm]<br>Enable Region Of Interest = false<br>Enable Region Growing = true<br>Enable Tracking = true<br>Enable Classify = false<br>Enable Shortest Distance = true<br>[Segmentation Setup]<br>Source Channel Index = 1<br>Enable Smooth = true<br>Surface Grain Size = 0.600 µm<br>[Machine Learning Training]<br>All Channels = false<br>[Split]<br>Region Growing Estimated Diameter = 12.0 µm<br>[Filter Seed Points]<br>"Quality" above 0.0500<br>[Filter Surfaces]<br>"Area" above 330 um^2<br>"Intensity StdDev Ch=1 Img=1" below 3.00<br>"Intensity Mean Ch=1 Img=1" between 1.00 and 8.50<br>[Tracking]<br>Algorithm Name = Brownian Motion<br>MaxDistance = 10.0 µm<br>MaxGapSize = 3<br>Fill Gap Enable = false<br>[Filter Tracks]	[Algorithm]<br>Enable Region Of Interest = false<br>Enable Region Growing = true<br>Enable Tracking = true<br>Enable Classify = false<br>Enable Shortest Distance = true<br>[Segmentation Setup]<br>Source Channel Index = 1<br>Enable Smooth = true<br>Surface Grain Size = 0.700 µm<br>[Machine Learning Training]<br>All Channels = false<br>[Split]<br>Region Growing Estimated Diameter = 15.0 µm<br>[Filter Seed Points]<br>"Quality" above 0.0250<br>[Filter Surfaces]<br>"Volume" above 1700 um^3<br>"Intensity Median Ch=1 Img=1" between 2.00 and 180.0<br>[Tracking]<br>Algorithm Name = Brownian Motion<br>MaxDistance = 10.0 µm<br>MaxGapSize = 2<br>Fill Gap Enable = false<br>[Filter Tracks]	[Algorithm]<br>Enable Region Of Interest = false<br>Enable Region Growing = true<br>Enable Tracking = false<br>Enable Classify = true<br>Enable Region Growing = true<br>Enable Shortest Distance = true<br>[Source Channel]<br>Source Channel Index = 2<br>Estimated XY Diameter = 0.900 µm<br>Estimated Z Diameter = 1.80 µm<br>Background Subtraction = true<br>[Filter Spots]<br>"Quality" above 1.75<br>"Shortest Distance to Surfaces Surfaces=**Cell Surface (STAb or Gal3)"** below 0.00<br>[Spot Region Type]<br>Region Growing Type = Local Contrast<br>[Spot Regions]<br>Region Growing Automatic Treshold = false<br>Region Growing Manual Threshold = 0.1<br>Region Growing Diameter = Diameter From Border<br>Create Region Channel = false<br>[Classification]<br>Group Name = PMProxClass<br>Input = All Spot<br>No. of Classes = 2<br>Class:: Name = PMProxSTEV<br>Class:: Name = PMDistSTEV<br>FilterType = Filter1D<br>Type = Shortest Distance to Surfaces<br>Surfaces=CellSurfaceGal3 JE-Gal3batchFinal.icsx_[ibrx_2025-06-13T14-50-49.726]<br>Threshold 1 = -0.500	[Algorithm]<br>Enable Region Of Interest = false<br>Enable Region Growing = true<br>Enable Tracking = false<br>Enable Classify = false<br>Enable Region Growing = true<br>Enable Shortest Distance = true<br>[Source Channel]<br>Source Channel Index = 1<br>Estimated XY Diameter = 0.850 µm<br>Estimated Z Diameter = 1.70 µm<br>Background Subtraction = true<br>[Filter Spots]<br>"Quality" above 3.50<br>"Intensity Max Ch=1 Img=1" above 20.0<br>"Shortest Distance to Surfaces Surfaces=**STAb cell Surface**" below 0.00 um<br>[Spot Region Type]<br>Region Growing Type = Local Contrast<br>[Spot Regions]<br>Region Growing Automatic Treshold = false<br>Region Growing Manual Threshold = 0.1<br>Region Growing Diameter = Diameter From Border<br>Create Region Channel = false	[Algorithm]<br>Enable Region Of Interest = false<br>Enable Region Growing = true<br>Enable Tracking = false<br>Enable Classify = false<br>Enable Region Growing = true<br>Enable Shortest Distance = true<br>[Source Channel]<br>Source Channel Index = 2<br>Estimated XY Diameter = 0.850 µm<br>Estimated Z Diameter = 1.70 µm<br>Background Subtraction = true<br>[Filter Spots]<br>"Quality" above 35.0<br>"Intensity Max Ch=2 Img=1" above 20.0<br>"Intensity StdDev Ch=2 Img=1" above 35.0<br>"Shortest Distance to Surfaces Surfaces=**Gal3 cell Surface**" below 0.00 um<br>[Spot Region Type]<br>Region Growing Type = Local Contrast<br>[Spot Regions]<br>Region Growing Automatic Treshold = false<br>Region Growing Manual Threshold = 0.1<br>Region Growing Diameter = Diameter From Border<br>Create Region Channel = false	preproc backgr filterwidth 0.3<br>[Algorithm]<br>Enable Region Of Interest = false<br>Enable Region Growing = false<br>Enable Tracking = false<br>Enable Classify = false<br>Enable Shortest Distance = false<br>[Segmentation Setup]<br>Source Channel Index = 2<br>Enable Smooth = true<br>Surface Grain Size = 0.130 µm<br>Enable Eliminate Background = false<br>[Threshold]<br>Active Threshold = true<br>Enable Automatic Threshold = false<br>Manual Threshold Value = 7.5<br>Active Threshold B = false<br>[Filter Surfaces]<br>"Number of Voxels Img=1" above 10.0	preproc backgr filterwidth 0.3<br>[Algorithm]<br>Enable Region Of Interest = false<br>Enable Region Growing = false<br>Enable Tracking = false<br>Enable Classify = false<br>Enable Shortest Distance = false<br>[Segmentation Setup]<br>Source Channel Index = 2<br>Enable Smooth = true<br>Surface Grain Size = 0.130 µm<br>Enable Eliminate Background = false<br>[Threshold]<br>Active Threshold = true<br>Enable Automatic Threshold = false<br>Manual Threshold Value = 2<br>Active Threshold B = false<br>[Filter Surfaces]<br>"Number of Voxels Img=1" above 10.0

**NLS-BFP Surface**<br>[Algorithm]<br>Enable Region Of Interest = false<br>Enable Region Growing = true<br>Enable Tracking = false<br>Enable Classify = false<br>Enable Shortest Distance = false<br>[Segmentation Setup]<br>Source Channel Index = 2<br>Enable Smooth = true<br>Surface Grain Size = 0.250 µm<br>[Machine Learning Training]<br>All Channels = false<br>[Split]<br>Region Growing Estimated Diameter = 12.0 µm<br>[Filter Seed Points]<br>[Filter Surfaces]<br>"Number of Voxels Img=1" above 10.0
