# Supplementary material for: Development of a Live‐Cell Imaging Assay to Elucidate Spatiotemporal Dynamics of Extracellular Vesicle Fusion with Target Cells
Source: J Extracell Vesicles. 2026 Mar 1;15(3):e70228. doi: 10.1002/jev2.70228 (PMC12949999; doi:10.1002/jev2.70228)
Supplement: Supplementary file 4 — Supporting Table 2: jev270228‐sup‐0004‐Table2.pdf [file JEV2-15-e70228-s004.pdf]

Requirement	Please Include Requested Information
1.1. Purpose	To assess the distribution of CFSE-stained extracellular vesicles (EVs) in density gradients in relation to Western blot data, in order to corroborate EV-associated release of SunTag from HeLa palm-mScar3-10xST cells. Additionally, to assess the relative concentrations of purified EV-preparations isolated from differently treated HeLa WT and palm-mScar3-10xST cells, in order to normalize the amount of EVs added to recipient cells in live-cell imaging experiments across conditions and experiments.
1.2. Keywords	Extracellular vesicles; EVs.
1.3. Experiment variables	WT and genetically engineered cells; mock- or VSV-G-transfected cells.
1.4. Organization name and address	Utrecht University, Yalelaan 1, Utrecht, The Netherlands.
1.5. Primary contact name and email address	Jasper van den Ende, j.vandenende@uu.nl (first author)<br>Esther Nolte-'t Hoen, e.n.m.nolte@uu.nl (senior author)
1.6. Date or time period of experiment	2024 – 2025
1.7. Conclusions	We developed a highly sensitive imaging method to detect EV-fusion events in real time, which has the power to illuminate the underexplored spatiotemporal dynamics of EV-fusion.
1.8. Quality control measures	Buffer noise reduction; reduction of unbound fluorescent dye; optimization of sample dilution; equal volume analysis for relative particle concentration comparison.
2.1.1.1. (2.1.2.1., 2.1.3.1.) Sample description	Extracellular vesicles isolated from cell culture supernatants, present in individual Optiprep density gradient fractions or as a purified preparation derived from pooled density gradient fractions.
2.1.1.2. Biological sample source description	HeLa R19 (human cervical carcinoma, ATCC CCL-2) and derivative transgenic HeLa palm-mScar3-10xST cell lines.
2.1.1.3. Biological sample source organism description	*Homo sapiens* (human), cervical adenocarcinoma.
2.1.2.2. Environmental sample location	N.A.
2.3. Sample treatment description	EVs were isolated from untreated or pCMV-VSVG-mScar3-10xST-transfected HeLa R19 cells, or from untreated or mock-transfected or pCMV-VSVG-transfected HeLa palm-mScar3-10xST cells.
2.4. Fluorescence reagent(s) description	CFSE (carboxyfluorescein succinimidylester)
3.1. Instrument manufacturer	Cytek
3.2. Instrument model	Aurora
3.3. Instrument configuration and settings	3L 16V-14B-8R, with Enhanced Small Particle (ESP) detection configuration
4.1. List-mode data files	N.A.
4.2. Compensation description	N.A.
4.3. Data transformation details	Events detected inside the EV-gate for PBS were subtracted from the events detected for other samples, to compensate for the buffer background.
4.4.1. Gate description	Representative images of gating strategy, as applied to all conditions, are shown in the associated publication. Gating was either on SSC/CFSE axes, or on SSC/FSC axes, depending on the experiment.
4.4.2. Gate statistics	See publication.
4.4.3. Gate boundaries	See publication.

Notes

Feel free to use more space than allocated.

You can embed graphics/figures in this document, if needed.
