# Supplementary material for: Development of a Live‐Cell Imaging Assay to Elucidate Spatiotemporal Dynamics of Extracellular Vesicle Fusion with Target Cells
Source: J Extracell Vesicles. 2026 Mar 1;15(3):e70228. doi: 10.1002/jev2.70228 (PMC12949999; doi:10.1002/jev2.70228)
Supplement: Supplementary file 6 — Supporting Figure 1: jev270228‐sup‐0006‐SuppMat.docx [file JEV2-15-e70228-s002.docx]

*Supplementary legends*

**Suppl. Figure 1. Density gradient distribution of EVs.** Refractive indices of individual density gradient fractions were measured using a refractometer and converted into density values. **(B)** Dot plots showing the CFSE fluorescence vs side-scatter (SSC) profiles of PBS only and EVs in fraction 8, with representative gating strategy. **(C)** EV-concentration as measured in the gate as set in (B) across fractions 4 through 11, corrected for dilution factors to yield the EV-concentration in the fractions. Graph shows the mean EV-concentration ± SD, calculated from n=2 independent experiments.

**Suppl. Figure 2. EV concentration measurements in purified EV preparations. (A)** Dot plots showing the forward (FSC) vs side-scatter (SSC) profiles of PBS only and gradient-purified EVs isolated from the indicated cell conditions, with representative gating strategy. **(B)** EV-concentration as measured in the gate as set in (A), corrected for dilution factors to yield the EV-concentrations in the preparations. Graph shows the mean EV-concentration ± SD, calculated from n=6-12 independent experiments. ** p≤0.01 and **** p≤0.0001 as determined by one-way ANOVA with Tukey’s multiple comparisons test.

**Suppl. Figure 3. Palm-mScar3-10xST recruits STAb-GFP upon co-expression. (A)** Live HeLa STAb-GFP cells were subjected to live-cell imaging after overnight mock- or palm-mScar3-10xST transfection. Images shown are maximum intensity projections, showing fluorescence for mScar3 (red) and GFP (green). Scale bars represent 10 µm.

**Suppl. Figure 4. Live-cell imaging of HeLa STAb-GFP cells after addition of SunTagged EVs allows for tracing of EV-binding, -uptake and -fusion over time – extended data. (A,B)** Additional timepoints of the data shown in Fig. 4B. Images are maximum intensity projections acquired at the indicated timepoints post-EV-addition, showing fluorescence channels for mScar3 **(A)** and GFP **(B)** in the same fields-of-view. Gamma was adjusted to 2 (mScar3) or 1.2 (GFP) for visualization purposes only. Scale bars represent 20 µm. Images are representative of n=3-4 independent experiments.

**Suppl. Figure 5. Differential segmentation allows for separate quantification of EV-binding and -uptake. (A)** Schematic overview of differential segmentation procedure. See Materials & Methods for detailed description. Created in BioRender. van den Ende, J. (2025) [https://BioRender.com/ycxyw00](https://biorender.com/ycxyw00). **(B)** 3D rendering of cell surface as segmented in Imaris, for a single STAb-GFP cell from the experiment also shown in Fig. 4. Cell boundary is depicted in gray. Differentially segmented mScar3 spots are shown in red, with a distance of <500 nm (EV-binding, left) or >500 nm (EV-uptake, right) to the segmented cell surface. Scale bars represent 5 µm. **(C-E)** Quantification of the data shown in Fig. 5 incorporating differential segmentation for EV-binding and -uptake, corrected for differences to the average cell volume per field-of-view. Graphs show the corrected mean mScar3 spots per timepoint **(C,D)** or mean maximum spot detection over the course of the experiment **(E)** per field-of-view for EV-binding or EV-uptake ± SEM, calculated from n=3-4 independent experiments with 3-6 fields-of-view per condition each.

**Suppl. Figure 6. STAb-GFP localization post-EV-fusion. (A)** Schematic depiction of observed post-fusion depletion of STAb-GFP from the nucleus. As described before, sequestration of STAb-GFP by SunTag in the cytosol results in a relative depletion of the nuclear signal, as the large complexes formed by interaction of multiple STAb molecules with the SunTagged molecule prohibits shuttling through nuclear pores. Created in BioRender. van den Ende, J. (2025) [https://BioRender.com/5yc11ya](https://biorender.com/5yc11ya). **(B)** Experimental data supporting model in (A). Live HeLa STAb-GFP cells expressing a nuclear localization signal-tagged blue fluorescent protein (NLS-BFP) were subjected to timelapse imaging immediately after addition of medium only or medium containing the indicated concentrations and types of EVs, taking Z-stacks at a 1h time interval. Images are mid-nuclear Z-slices acquired at 6h post-EV-addition, showing fluorescence channels for GFP **(top)** and BFP **(middle)** in the same fields-of-view. Scale bars represent 5 µm. **(C)** After segmentation of cells in 3D based on the GFP channel, a nuclear volume was segmented based on the BFP channel. GFP fluorescent intensity was measured in both the total cell and nuclear volumes. Graph shows the calculated ratio of the mean nuclear intensity to the mean total cell intensity ± SD, calculated from 6 fields-of-view per condition. **(D)** Schematic depiction of observed post-fusion localization of STAb-GFP, reflecting the sorting of the SunTagged EV-associated molecule. When using palm-10xST EVs which are additionally mounted with VSV-G, as described in the rest of the data, STAb-GFP puncta eventually disappear. This is likely the result of a balance between palmitoylation and depalmitoylation in the target cell, resulting in diffusion of STAb-SunTag complexes into the cytosol. Eventually, these complexes can be seen to be re-inserted at the plasma membrane. When using EVs carrying directly SunTagged VSV-G, STAb-GFP remains punctate post-fusion, indicating it recycles through the endosomal system as is known for VSV-G, eventually concentrating at the plasma membrane. Created in BioRender. van den Ende, J. (2025) [https://BioRender.com/3rw27gl](https://biorender.com/3rw27gl). **(E)** Experimental data supporting models in (D). Live HeLa STAb-GFP cells were subjected to timelapse imaging immediately after addition of medium containing EVs isolated from VSV-G-transfected HeLa palm-mScar3-10xST cells, or from VSV-G-mScar3-10xST-transfected HeLa WT cells. Images are mid-nuclear Z-slices acquired at the indicated times post-EV-addition, showing GFP fluorescence. Scale bars represent 5 µm.

**Suppl. Figure 7. EV-FUSIM allows for tracing of EV-binding, -uptake and –fusion over time in A549 STAb-GFP recipient cells. (A)** Live A549 STAb-GFP cells were subjected to timelapse imaging immediately after addition of medium only or medium containing the indicated concentrations and types of EVs, taking Z-stacks at a 1h time interval. Images are maximum intensity projections acquired at 2h post-EV-addition, showing fluorescence channels for mScar3 **(top)** and GFP **(middle)** in the same fields-of-view. Gamma was adjusted to 2 (mScar3) or 1.2 (GFP) for visualization purposes only. Scale bars represent 20 µm. White insets correspond to magnifications **(bottom)**, which show mScar3, GFP and merged channels. Scale bar represents 5 µm. Images are representative of n=3 independent experiments. **(B-E)** After segmentation of cells in 3D based on the GFP channel, cell-associated fluorescent spots in the red and green channel were counted for each field-of-view. In tandem, the total cell volume per field-of-view was counted, which was used to correct spot counts for differences to the average cell volume per field-of-view. Graphs show the corrected mean spots per timepoint **(B, D)** or mean maximum spot detection over the course of the experiment **(C, E)** per field-of-view for both mScar3 and GFP channels ± SEM, calculated from n=3 independent experiments with 4-6 fields-of-view per condition each. * p≤0.05 and ** p≤0.01 as determined by one-way ANOVA with Tukey’s multiple comparisons test.

**Suppl. Figure 8. Down-titration of VSV-G EVs reveals significant detection of EV-binding/uptake and –fusion until 125 EVs/cell. (A**) Live HeLa STAb-GFP cells were subjected to timelapse imaging immediately after addition of medium only or medium containing the indicated concentrations of VSV-G EVs, taking Z-stacks at a 15 min time interval. Images are maximum intensity projections acquired at 1h post-EV-addition, showing fluorescence channels for mScar3 **(top)** and GFP **(middle)** in the same fields-of-view. Gamma was adjusted to 2 (mScar3) or 1.2 (GFP) for visualization purposes only. Scale bars represent 20 µm. White insets correspond to magnifications **(bottom)**, which show mScar3, GFP and merged channels. Scale bar represents 5 µm. Images are representative of n=3 independent experiments. **(B-E)** After segmentation of cells in 3D based on the GFP channel, cell-associated fluorescent spots in the red and green channel were counted for each field-of-view. In tandem, the total cell volume per field-of-view was counted, which was used to correct spot counts for differences to the average cell volume per field-of-view. Graphs show the corrected mean spots per timepoint **(B, D)** or mean maximum spot detection over the course of the experiment **(C, E)** per field-of-view for both mScar3 and GFP channels ± SEM, calculated from n=3 independent experiments with 3-4 fields-of-view per condition each. * p≤0.05 as determined by unpaired t-test, compared to the medium control.

**Suppl. Figure 9. VSV-G-mediated EV-fusion occurs in the absence of endolysosomal rupture – extended data. (A)** Schematic of Galectin-3 reporter assay principle. HeLa cells were engineered to express mAzamiGreen-tagged Galectin-3, which is present abundantly in the cytosol and has strong affinity for carbohydrate moieties. Many of these are present inside the endosomal lumen, but this pool remains inaccessible for cytosolic Gal3 unless there is rupture of the endosomal membrane. Upon addition of VSV-G EVs, red spots will indicate EV-binding/uptake, whereas green spots will indicate endolysosomal rupture. As we know SunTag is exposed to the cytosol from these EVs, absence of endolysosomal rupture detection will further prove fusion as the responsible mechanism. Created in BioRender. van den Ende, J. (2025) [https://BioRender.com/tm3vcwj](https://biorender.com/tm3vcwj). **(B)** Images are maximum intensity projections acquired at 1h post-EV-addition, showing fluorescence for mScar3 in the same fields-of-view as shown in Fig. 6H. Gamma was adjusted to 2 (mScar3) for visualization purposes only. Scale bars represent 20 µm. Images are representative of n=3-4 independent experiments. **(C, D)** After segmentation of cells in 3D based on the mAG channel, cell-associated fluorescent spots in the red channel were counted for each field-of-view. In tandem, the total cell volume per field-of-view was counted, which was used to correct spot counts for differences to the average cell volume per field-of-view. Graph shows the corrected mean spots per timepoint **(H)** or mean maximum spot detection over the course of the experiment **(I)** per field-of-view for the mScar3 channel ± SEM, calculated from n=3 independent experiments with 5-6 fields-of-view per condition each. **** p≤0.0001 as determined by one-way ANOVA with Tukey’s multiple comparisons test.

**Suppl. Figure 10. Separate quantification of EV-binding and –uptake confirms BafA1 treatment and VSV-G P127D mutagenesis did not affect either. (A)** Quantification of the data shown in Fig. 7 incorporating differential segmentation for EV-binding and -uptake, corrected for differences to the average cell volume per field-of-view. Graphs show the corrected mean maximum mScar3 spot detection over the course of the experiment per field-of-view for EV-binding or EV-uptake ± SEM, calculated from n=3-4 independent experiments with 4-6 fields-of-view per condition each. ns p>0.05 as determined by as determined by unpaired t-test (performed separately for binding and uptake). **(B)** Quantification of the data shown in Fig. 8 incorporating differential segmentation for EV-binding and -uptake, corrected for differences to the average cell volume per field-of-view. Graphs show the corrected mean maximum mScar3 spot detection over the course of the experiment per field-of-view for EV-binding or EV-uptake ± SEM, calculated from n=3 independent experiments with 4 fields-of-view per condition each. ns p>0.05, * p≤0.05 and ** p≤0.01 as determined by one-way ANOVA with Tukey’s multiple comparisons test (performed separately for binding and uptake).

**Suppl. Figure 11. Transfection of HeLa palm-mScar3-10xST cells with VSV-G P127D results in release of VSV-G P127D-containing SunTagged EVs. (A)** Mock- , VSV-G WT- or VSV-G P127D-transfected HeLa palm-mScar3-10xST cells were fixed after overnight transfection followed by 24h incubation in EV-depleted medium, then immunolabeled for VSV-G. Images shown are mid-nuclear Z-slices, showing fluorescence for mScar3 (red), VSV-G (green) and Hoechst-labeled nuclei (white). Scale bars represent 10 µm. **(B)** EV-containing ultracentrifugation pellets from VSV-G P127D-transfected cells were floated into iodixanol density gradients, after which individual gradient fractions were concentrated using TCA precipitation and analyzed for the presence of protein markers by Western blotting. **(C)** EV-concentrations in gradient-purified EV preparations isolated from mock-, VSV-G WT- or VSV-G P127D-transfected cells were quantified using high resolution flow cytometry. Graph shows the mean fold change to transfection ctl EVs ± SD, calculated from n=4 independent experiments. * p≤0.05 and ** p≤0.01 as determined by one-way ANOVA with Tukey’s multiple comparisons test.

**Suppl. Figure 12. Localization experimental procedures did not alter VSV-G-mediated EV-fusion dynamics. (A, B)** Analysis of experimental conditions also shown in Fig. 8 & 9. After segmentation of cells in 3D based on the GFP channel, cell-associated fluorescent spots in the green channel were counted for each field-of-view. In tandem, the total cell volume per field-of-view was counted, which was used to correct spot counts for differences to the average cell volume per field-of-view. Graphs show the corrected mean spots per timepoint **(A)** or mean maximum spot detection over the course of the experiment **(B)** per field-of-view for the GFP channel ± SEM, calculated from n=3-4 independent experiments with 4-6 fields-of-view per condition each. ns p>0.05 as determined by one-way ANOVA with Tukey’s multiple comparisons test.

**Suppl. Video 1. EV-FUSIM facilitates real-time visualization of VSV-G-mediated EV-fusion.** Timelapse video of data also shown in Fig. 6. Live HeLa STAb-GFP cells were subjected to timelapse imaging immediately after addition of medium containing 10^8^ VSV-G EVs, taking Z-stacks at a 15 min time interval. Images are maximum intensity projections acquired at a 15 min time interval, showing fluorescence channels for mScar3 **(left)** and GFP **(right)** in the same fields-of-view. Scale bars represent 20 µm. Time after EV-addition is indicated as hh:mm.

**Suppl. Table 1. Imaris object creation scripts.** Shown are the parameters used in Imaris to generate the denoted 3D objects, used for downstream analysis and quantification.

**Suppl. Table 2. Completed MIFlowCyt checklist.** Contains a completed checklist reporting on experimental variables related to flow cytometry experiments.

**Suppl. Table 3. Completed MIFlowCyt-EV checklist.** Contains an additional completed checklist reporting on additional experimental variables related to EV-flow cytometry experiments.
